# Supplementary material for: RasGRP1 promotes the acute inflammatory response and restricts inflammation-associated cancer cell growth
Source: Nat Commun. 2022 Nov 16;13:7001. doi: 10.1038/s41467-022-34659-x (PMC9669001; doi:10.1038/s41467-022-34659-x)
Supplement: Supplementary file 1 — Supplementary Information [file 41467_2022_34659_MOESM1_ESM.pdf]

Supplementary Information

RasGRP1 promotes the acute inflammatory response and restricts inflammation-associated cancer cell growth

Cong Wang[1,5], Xue Li[1,5], Binbin Xue[1,5], Changping Yu[1,5], Luoling Wang[1,5], Rilin Deng[1], Hui Liu[1], Zihao Chen[1], Yingdan Zhang[1], Suping Fan[2,3], Chaohui Zuo[4], Hungyu Sun[1], Haizhen Zhu[1*], Jianli Wang[2,3*], Songqing Tang[1*]

[1]Institute of Pathogen Biology and Immunology, Department of Pharmacy, College of Biology, Hunan Provincial Key Laboratory of Medical Virology, Hunan University, Changsha 410082, China

[2]Institute of Immunology, and Bone Marrow Transplantation Center of the First Affiliated Hospital, Zhejiang University School of Medicine, Hangzhou 310058, China

[3]Institute of Hematology, Zhejiang University & Zhejiang Engineering Laboratory for Stem Cell and Immunotherapy, Hangzhou 310058, China

[4]Department of Gastroduodenal and Pancreatic Surgery, Translational Medicine Research Center of Liver Cancer, Hunan Cancer Hospital, Changsha 410013, China

[5]Cong Wang, Xue Li, Binbin Xue, Changping Yu and Luoling Wang contributed equally to this work.

Correspondence: Haizhen Zhu (zhuhaizhen69@yahoo.com), Jianli Wang (jlwang@zju.edu.cn) or Songqing Tang (tangsq@hnu.edu.cn)

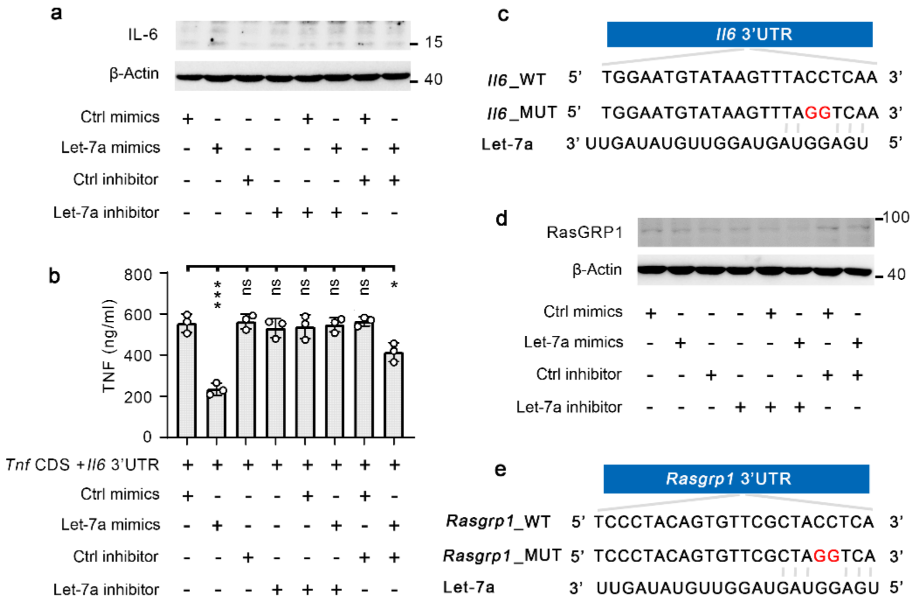

Supplementary Figure 1. *Il6* and *Rasgrp1* expression levels are regulated by let-7a. **a** Immunoblot showing IL-6 in lysates of HEK293 cells transfected with the indicated molecules but not the *Il6* CDS for 24 h. **b** ELISA and quantification of Tnf in the supernatants of HEK293 cells transfected with the indicated molecules for 24 h (means ± SEM, One-way ANOVA Tukey test, ***p = 0.0001, *p = 0.012 in sequence, n = three independent experiments). **c** Mutant *Il6* 3'UTR, which carried a mutated sequence in the site that is complementary to the seed region of let-7a, was generated using the fusion PCR method. **d** Immunoblot showing Rasgrp1 in lysates of HEK293 cells transfected with the indicated molecules but not the *Rasgrp1* CDS for 24 h. **e** Mutant *Rasgrp1* 3'UTR, which carried a mutated sequence in the site complementary to the seed region of let-7a, was generated using the fusion PCR method. The data shown in **a** and **d** are from one representative experiments of three independent experiments. ns, not significant; * $p < 0.05$, ** $p < 0.01$, *** $p < 0.001$.

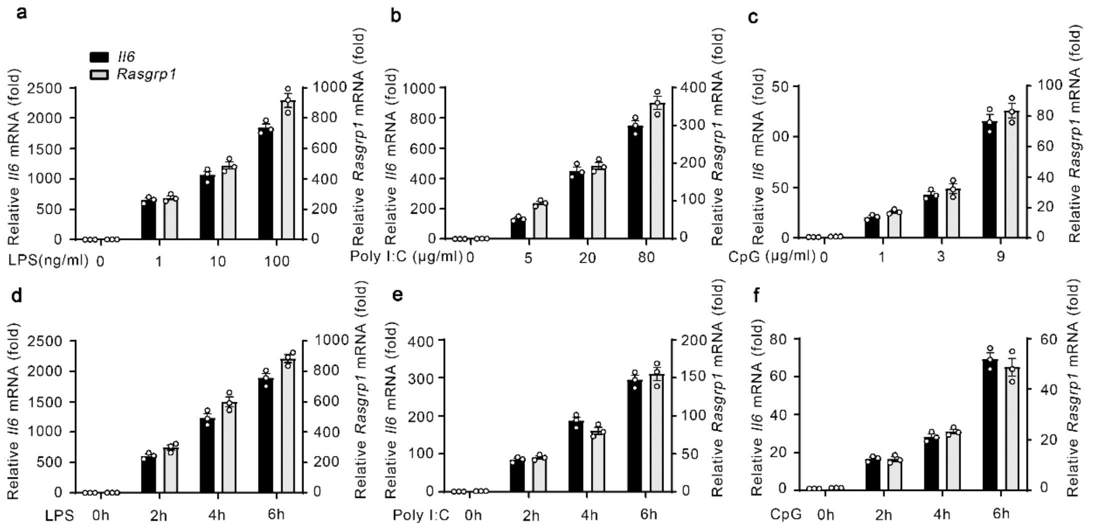

Supplementary Figure 2. *Rasgrp1* coexpressed with *Il6* in an acute inflammatory response. **a-c** qPCR analysis of *Il6* and *Rasgrp1* mRNA expression in bone marrow-derived macrophages treated with different doses of lipopolysaccharide (LPS; 0, 1, 10 or 100 ng/ml) (**a**), polyinosinic:polycytidylic acid (poly (I:C); 0, 5, 20 or 80 µg/ml) (**b**), or CpG oligodeoxynucleotide (ODN; 0, 1, 3 or 9 µg/ml) (**c**) for 6 h. **d-f** qPCR analysis of *Il6* and *Rasgrp1* mRNA expression in bone marrow-derived macrophages treated with 100 ng/ml LPS (**d**), 10 µg/ml poly (I:C) (**e**), or 5 µg/ml CpG ODN (**f**) for the indicated times (hours). The data are shown as mean ± SEM of three independent experiments (**a-f**).

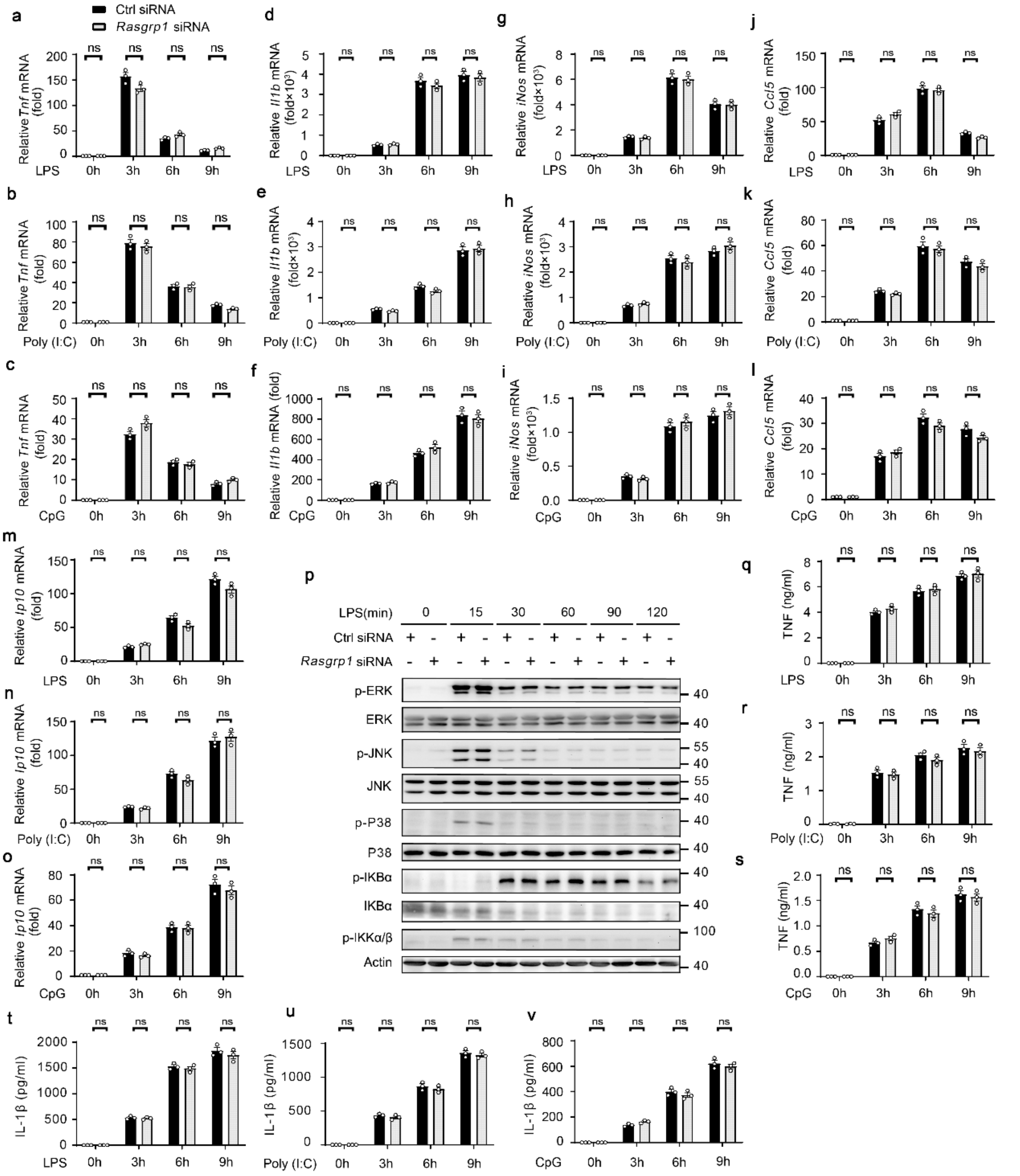

Supplementary Figure 3. ***Rasgrp1*** **silencing has no effect on the mRNA levels of cytokines in peritoneal macrophages**. **a-o** qPCR analysis of *Tnf* (**a-c**) *Il1b* (**d-f**), *iNos* (**g-i**) *Ccl5* (**j-l**) and *I10* (**m-o**) mRNA expression in peritoneal macrophages transfected with *Rasgrp1* siRNA and treated 48h later with lipopolysaccharide (LPS) (**a, d, g, j, m**), polyinosinic:polycytidylic acid (poly (I:C)) (**b, e, h, k, n**) or CpG oligodeoxynucleotide (ODN) (**c, f, I, l, o**) for 6 h. **p** Immunoblot analysis of the indicated molecules in the

lysates of peritoneal macrophages transfected with *Rasgrp1* siRNA and treated 48h later with 100 ng/ml LPS for the indicated times (minutes). **q-v** ELISA quantification of TNF (**q-s**) and IL-1β (**t-v**) in the supernatants of macrophages treated as described in (**a-f**). The data are shown as mean ± SEM of three independent experiments in (**a-o**) and (**q-v**). The data shown in **p** is from one representative experiments of three independent experiments. Statistical analysis was performed by paired t test, two-side. ns, not significant.

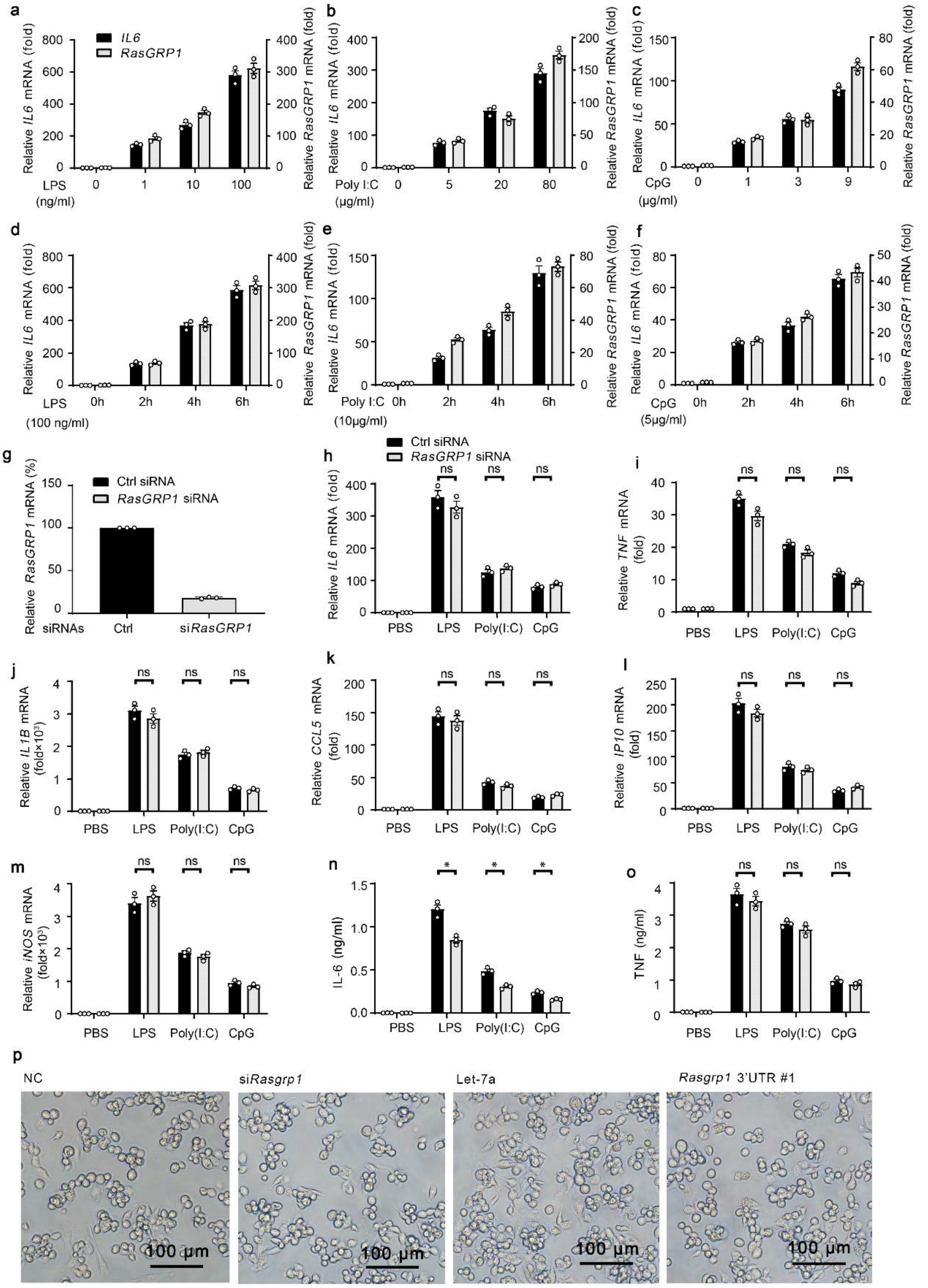

Supplementary Figure 4. *RasGRP1* coexpressed with *IL6*, and *RasGRP1* silencing selectively inhibits IL-6 protein levels in human peripheral blood monocyte-derived macrophages (MDMs). a-c qPCR analysis of *IL6* and *RasGRP1* mRNA

expression in human MDMs treated with different doses of lipopolysaccharide (LPS; 0, 1, 10 or 100 ng/ml) (**a**), polyinosinic:polycytidylic acid (Poly (I:C); 0, 5, 20 or 80 μg/ml) (**b**), or CpG oligodeoxynucleotide (ODN; 0, 1, 3 or 9 μg/ml) (**c**) for 6 h. **d-f** qPCR analysis of *IL6* and *RasGRP1* mRNA expression in human MDMs treated with 100 ng/ml LPS (**d**), 10 μg/ml poly (I:C) (**e**), or 5 μg/ml CpG ODN (**f**) for the indicated times (hours). **g** qPCR analysis of *RasGRP1* mRNA expression in human MDMs 48 h after transfection with *RasGRP1* short interfering RNA (siRNA). **h-m** qPCR analysis of *IL6* (**h**), *TNF* (**i**) *IL1B* (**j**), *CCL5* (**k**), *IP10* (**l**) and *iNOS* (**m**) mRNA expression in human MDMs transfected with *RasGRP1* siRNA and treated 48 h later with LPS, poly (I:C) or CpG ODN for 6 h. **n-o** ELISA and quantification of IL-6 (**n**) and TNF (**o**) in the supernatants of human MDMs transfected with *RasGRP1* siRNA and treated 48h later with LPS, poly (I:C) or CpG ODN for 6 h (means ± SEM, paired t test, one-sided, for **n**: *p=0.0391, two-sided, **p = 0.0021, **p = 0.0051 in sequence, n = 3 independent experiments). **p** Viability of primary macrophages after transfecting with negative control (NC), *Rasgrp1* siRNA, let-7a and *Rasgrp1* 3' UTR #1 for 3 days. (The data shown in **p** is from one representative experiments of three independent experiments). The data are shown as mean ± SEM of three independent experiments. Statistical analysis was performed by paired t test, two-side. ns, not significant; * $p < 0.05$.

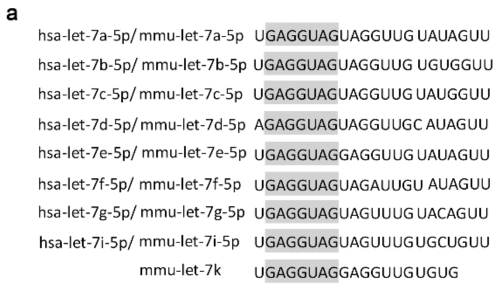

Supplementary Figure 5. Let-7 family members have a conserved seed sequence.

a Schematic diagram showing let-7 family members with conserved seed sequences in humans and mice.

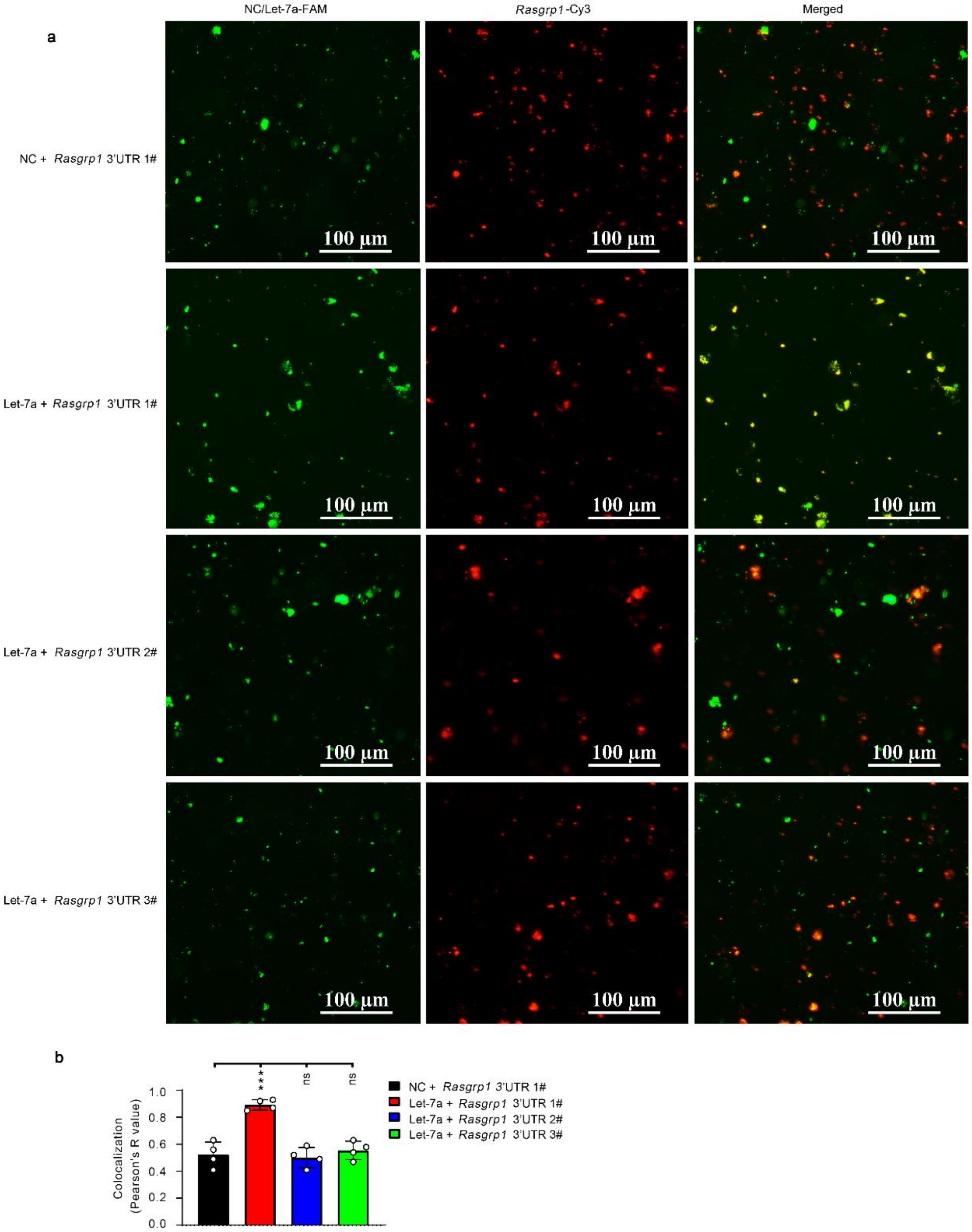

Supplementary Figure 6. Let-7a binds to the *Rasgrp1* 3'UTR. a Confocal microscopy image showing peritoneal macrophages cotransfected with negative control (NC)/let-7a-FAM (green) and its sponge RNA *Rasgrp1* 3'UTR #1, #2 or #3-Cy3 (red) (The data shown in **a** is from one representative experiments of three independent

experiments. **b** Colocalization analysis (**a**) with the Coloc 2 plug-in program. (The values are reported as the means ± SEM of four independent experiments, One-way ANOVA Tukey test, *** $p = 0.0001$)

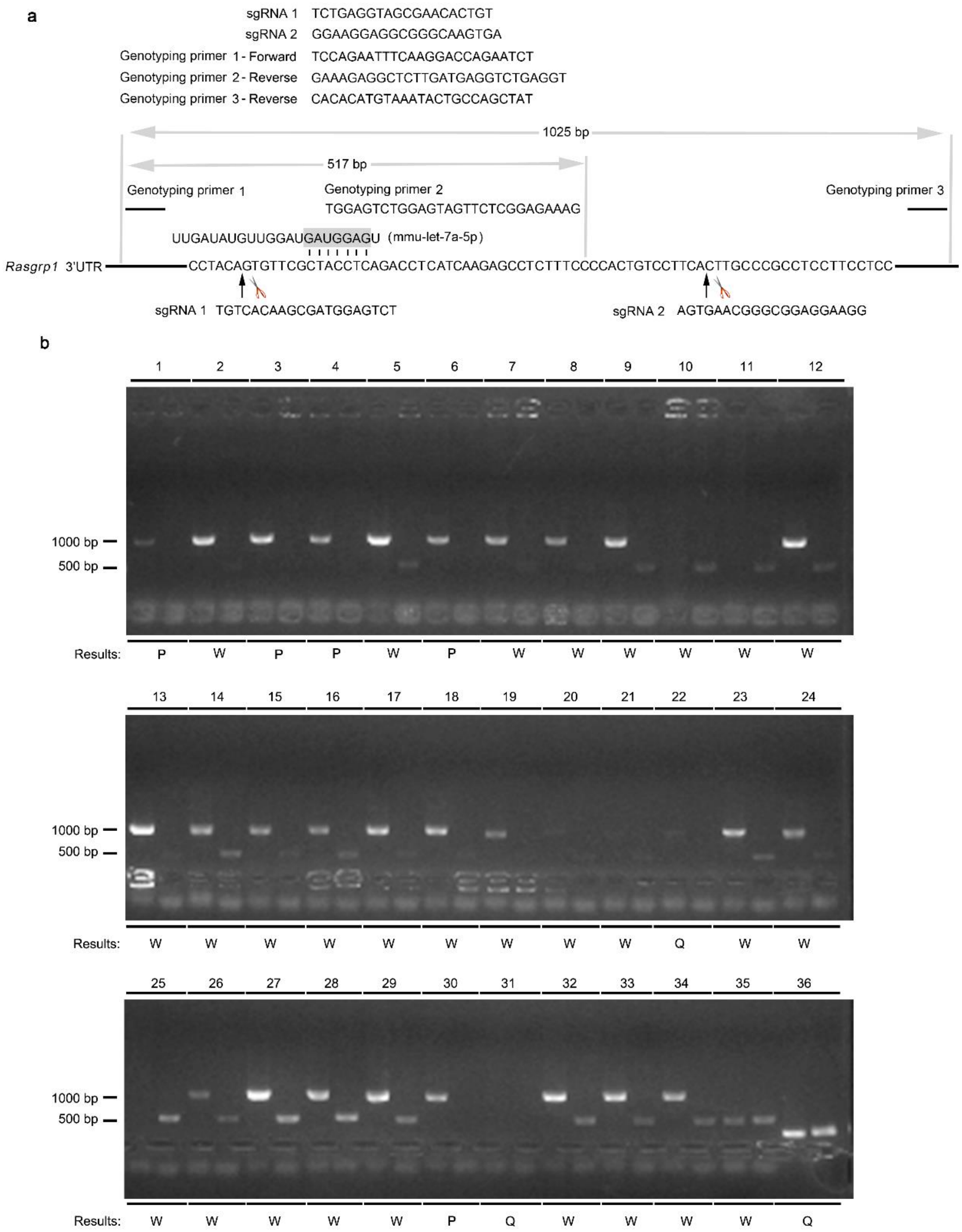

Supplementary Figure 7. ***Rasgrp1*** **3'UTR mutant clones were constructed and expressed in RAW264.7 cells**. **a** Schematic diagram showing the sequences of two single guide RNAs (sgRNAs) designed for knocking out the binding site of let-7a in the *Rasgrp1* 3'UTR and three genotyping primers designed for screening the targeted

mutation. **b** PCR screening of the targeted mutation in the *Rasgrp1* 3'UTR in the RAW264.7 macrophage clone. The PCR results were evaluated as indicated at the bottom (“P” indicates positive, “W” indicates wild type, and “Q” indicates issues with DNA quality). Only positive RAW264.7 macrophage clones were selected, and these clones were confirmed by sequencing.

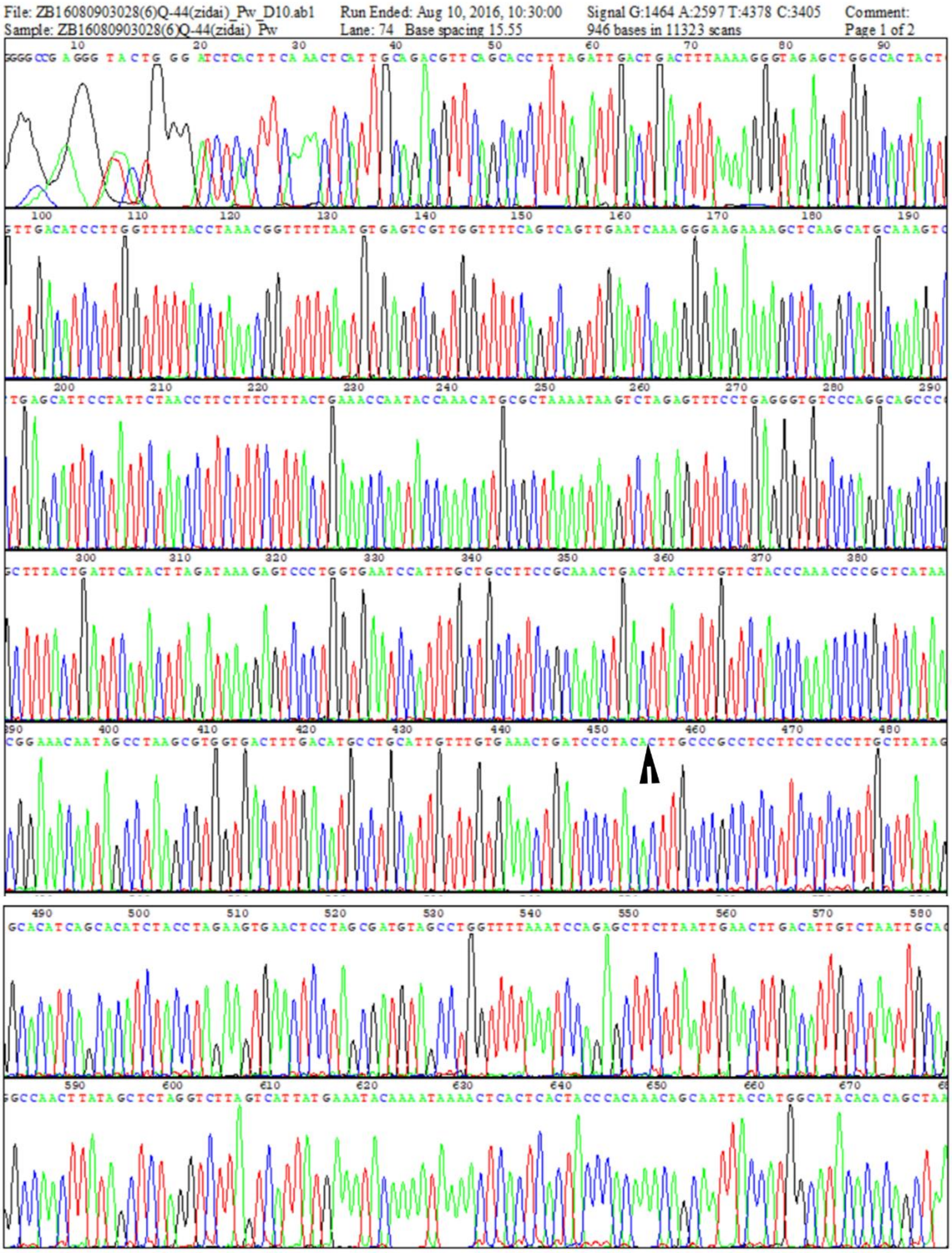

Supplementary Figure 8. DNA sequencing map of the mutant *Rasgrp1* 3'UTR. The black arrow points to the mutation site in the *Rasgrp1* 3'UTR in RAW264.7 macrophage clones. The mutation contains a 51-bp deletion that included the let-7a binding site.

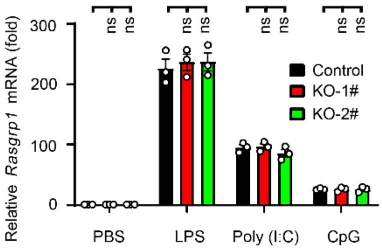

Supplementary Figure 9. *Rasgrp1* mRNA level was not affected by mutation of the *Rasgrp1* 3' UTR. qPCR analysis of *Rasgrp1* mRNA expression in RAW264.7 macrophage clones (KO-1# or KO-2# with a 51-bp deletion) treated with lipopolysaccharide (LPS), polyinosinic:polycytidylic acid (poly (I:C)) or CpG oligodeoxynucleotides (ODN) for 6 h (The values are reported as the means ± SEM of three independent experiments, One-way ANOVA LSD test). ns, not significant.

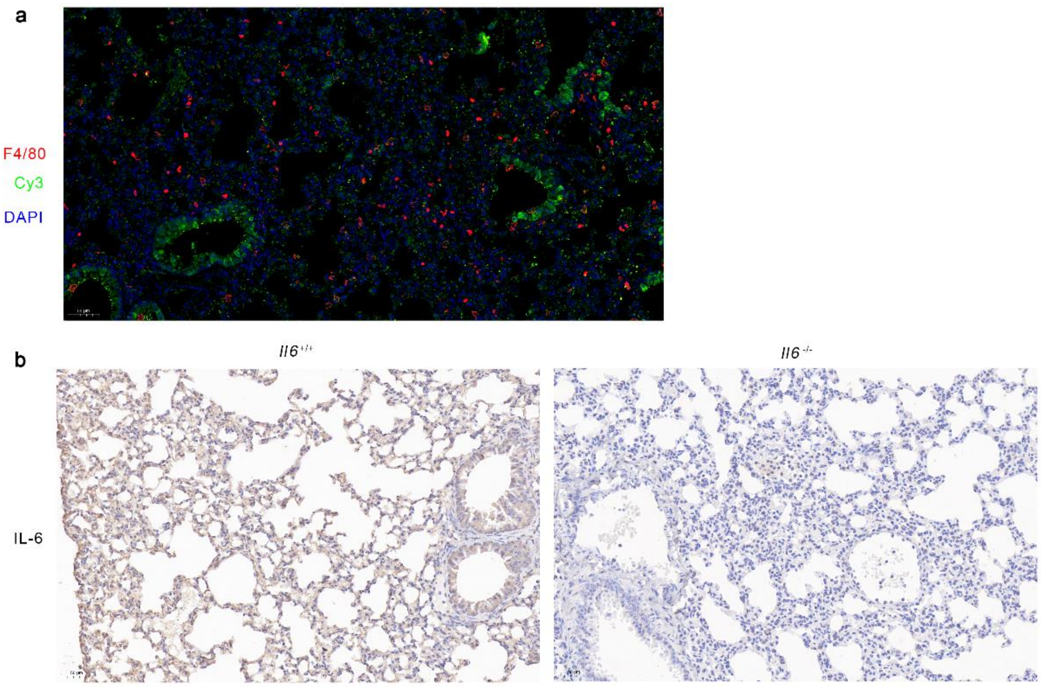

Supplementary Figure 10. a Immunofluorescence (IF) costaining for macrophages and Cy3 labelled *Rasgrp1* 3'UTR #1 in the lungs of *Il6*$^{+/+}$ mice treated by intravenous injection with Cy3-labelled *Rasgrp1* 3'UTR #1 and intraperitoneally injected with lipopolysaccharide (LPS) 24 h later; the analysis was performed 8 h after the LPS treatment. **b** Immunohistochemistry staining for IL-6 protein in the lungs of *Il6*$^{+/+}$ mice and *Il6*$^{-/-}$ mice treated with LPS for 8 h. The data shown in **a** and **b** are from one representative experiments of three independent experiments.

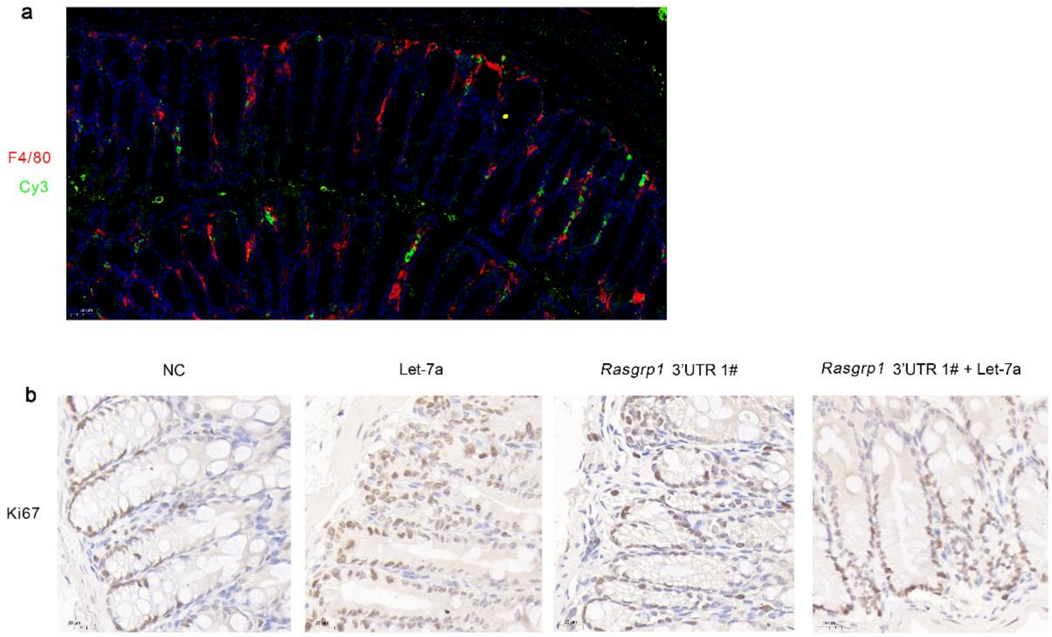

Supplementary Figure 11. a Immunofluorescence (IF) costaining for macrophages and Cy3 labelled *Rasgrp1* 3'UTR #1 in the colons of *Il6*$^{+/+}$ mice intravenously injected with Cy3-labelled *Rasgrp1* 3'UTR #1 every three days and fed with 2.5% DSS in water for 7 days. **b** Immunohistochemistry (IHC) staining for ki-67 in the colons of *Il6*$^{+/+}$ mice intraperitoneally injected with cholesterol-conjugated microRNA (miRNA) et-7a and/or its sponge RNA *Rasgrp1* 3'UTR #1 every three days and fed with 2.5% DSS in water for 7 days. The data shown in **a** and **b** are from one representative experiments of three independent experiments.

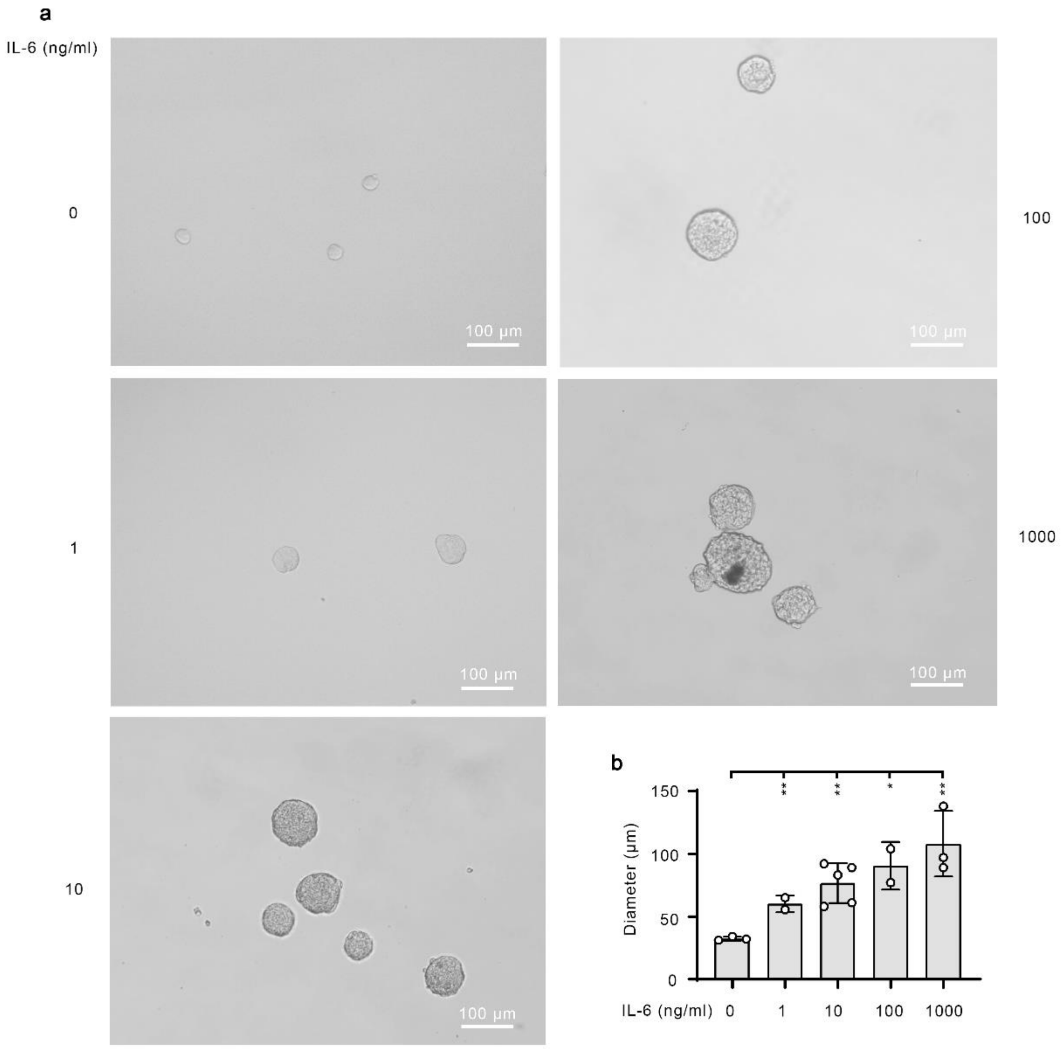

Supplementary Figure 12. IL-6 promotes the growth of liver cancer progenitors. **a** Huh 7 cells were cultured with 20 ng/ml EGF, 20 ng/ml FGF, B27 (2%) and the indicated concentrations of IL-6 on low-attachment plates for 7 days (The data show in **a** are from one representative experiment of three independent experiments). **b** Diameter of the hepatocellular carcinoma (HCC) progenitor cell-like spheroids shown in (**a**) (means ± SD, unpaired t test, two-sided, **p = 0.0051, **p = 0.0036, *p = 0.0105, **p = 0.0076, in sequence, n = 2-5 HCC progenitor cell-like spheroids).

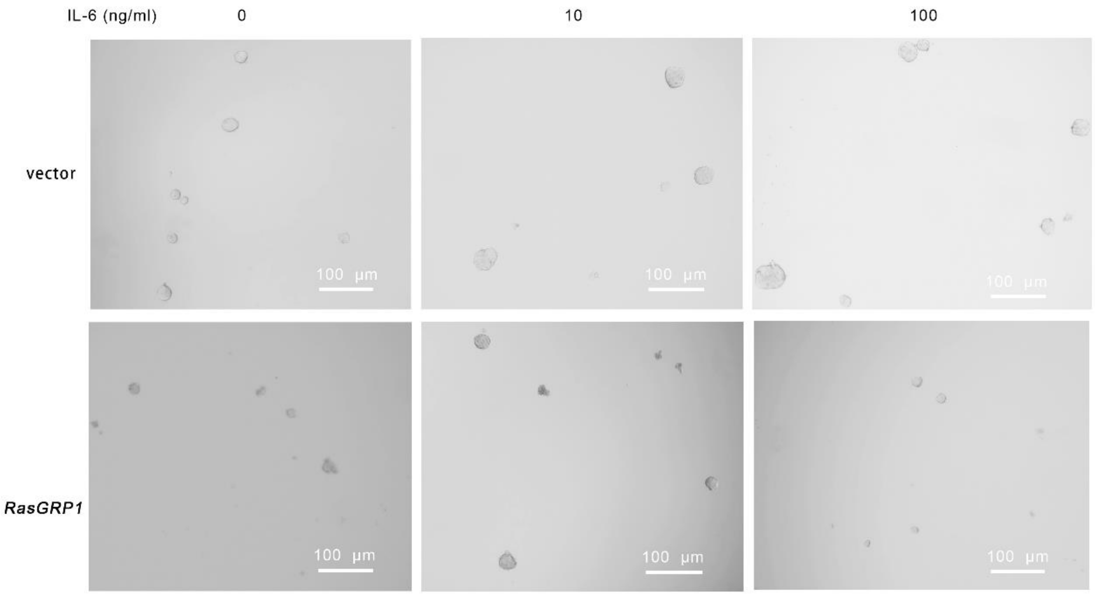

Supplementary Figure 13. RasGRP1 inhibits the growth of liver cancer progenitors. Huh7 cells with overexpressing lentivirus-delivered RasGRPP1 or not were cultured with 20 ng/ml EGF, 20 ng/ml FGF, B27 (2%) and the indicated concentrations of IL-6 on low-attachment plates for 7 days (Similar results were obtained from three independent experiments).

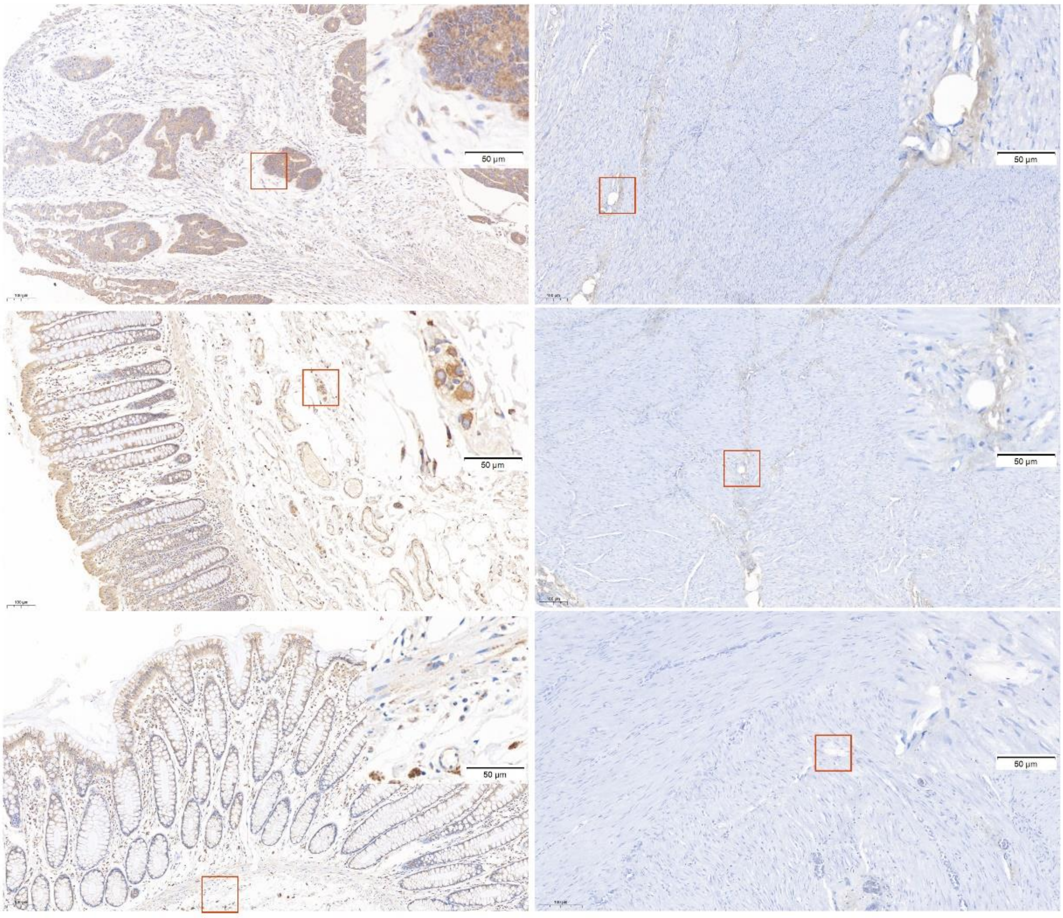

Supplementary Figure 14. Specificity of anti-RasGRP1 antibodies. Anti-RasGRP1 antibody immunohistochemistry (IHC) staining (Primary antibody: anti-RasGRP1, 1:100 dilution, Abcam, ab37927; Second antibody: HRP-labelled goat anti-rabbit IgG, 1:1000 dilution, Sigma-Aldrich, AP132P) of colons from colon cancer patients (n = 3, left) and gastric tissues from gastric cancer patients (n = 3, right).

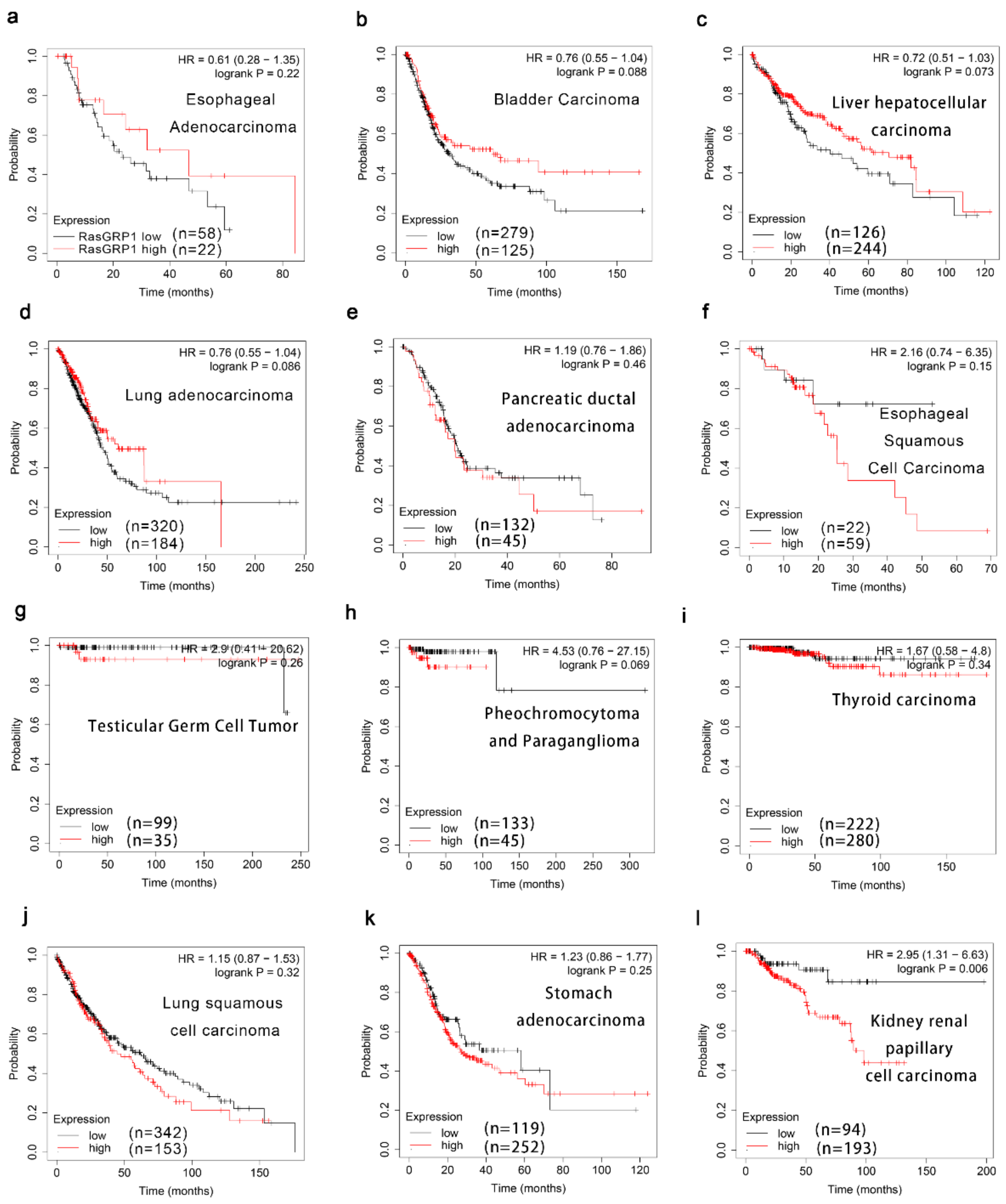

Supplementary Figure 15. RasGRP1 expression correlates with the overall survival of cancer patients. a-l The survival curves from Kaplan–Meier plot profiles (http://kmplot.com/analysis/) of cancer patients stratified by high and low RasGRP1 expression.

Supplementary Table 1. Primers used in vector construction.

Vector	Forward Primer (5'→3')	Reverse Primer (5'→3')
Mouse *Il6* (CDS+3'UTR)	GGCCTCGAGGCCATGAAGTTCCTCTCTGCAAG	TAAGGATCCAGCAATTCATTGAGGTAAACTTATA
Mouse *Rasgrp1* (CDS+3'UTR)	AATGCGGCCGCATGGGAACCCTGGGCAAGGCGAG	TAAGGATCCTGATGAGGTCTGAGGTAGCGAACACT
Human *RasGRP1* (3* Flag Vector)	CCCAAGCTTGCCATGGGCACCCTGGGCAAGGCGAGAG	CGGGATCCAGAACAGTCACCCTGCTCCATT
Mouse *Tnf*-CDS + *Il6*-3'UTR-1#	CGCGGATCCATGAGCACAGAAAGCATGATCCGCG	ATGAATTGGATGGTCTTGGTCCTCACAGAGCAATGACTCCAAAGTAG
Mouse *Tnf*-CDS + *Il6*-3'UTR-2#	CTACTTTGGAGTCATTGCTCTGTGAGGACCAAGACCATCCAATTCAT	CCGCTCGAGGGTTTTTAAATATAATATAATTTAT
Mouse *Rasgrp1* 3'UTR	CGCGGATCCTCCAGAATTTCAAGGACCAGAATCT	CCGCTCGAGGTGTATGCCATGGTAATTGCTGTTT
Mouse *Rasgrp1* 3'UTR mut-1#	CGCGGATCCTCCAGAATTTCAAGGACCAGAATCT	AGAGGCTCTTGATGAGGTCTGAGGTAGCGAACACTGTAGGGATCAGTTTCAC
Mouse *Rasgrp1* 3'UTR mut-2#	GTGAAACTGATCCCTACAGTGTTCGCTACCTCAGACCTCATCAAGAGCCTCT	CCGCTCGAGGTGTATGCCATGGTAATTGCTGTTT

Supplementary Table 2. Primers used in Q-PCR assays.

Gene Target	Forward Primer (5'→3')	Reverse Primer (5'→3')
Mouse *Rasgrp1*	ACTGCCACCTCATCGACAC	CACTTTGCGCTTCTTGCTAGTA
Mouse *Il6*	GAGTTGTGCAATGGCAATTCTG	GCAAGTGCATCATCGTTGTTCAT
Mouse *Edn1*	GCACCGGAGCTGAGAATGG	GTGGCAGAAGTAGACACACTC
Mouse *Nlrc5*	GCTGAGAGCATCCGACTGAAC	AGGTACATCAAGCTCGAAGCA
Mouse *Socs1*	CTGCGGCTTCTATTGGGGAC	AAAAGGCAGTCGAAGGTCTCG
Mouse *Olr1*	CAAGATGAAGCCTGCGAATGA	ACCTGGCGTAATTGTGTCCAC
Mouse *Xkr8*	GGCCGTTGTCCAGTACGTG	GCAAACACCTATACAGGTAGCC
Mouse *Tnfaip3*	GAACAGCGATCAGGCCAGG	GGACAGTTGGGTGTCTCACATT
Mouse *Etnk1*	CTGTTCACAGATGGGATCACAA	CGCGGAAACTTTTCACTTCCTC
Mouse *Cd86*	TGTTTCCGTGGAGACGCAAG	TTGAGCCTTTGTAAATGGGCA
Mouse *Parp8*	TAAATCGCACAAACTTTTGGGC	TCTCCAGAACAAGATCGAGTCAA
Mouse *Tnf*	CCCTCACACTCAGATCATCTTCT	GCTACGACGTGGGCTACAG
Mouse *Il1b*	GAAATGCCACCTTTTGACAGTG	CTGGATGCTCTCATCAGGACA
Mouse *iNos*	ACATCGACCCGTCCACAGTAT	CAGAGGGGTAGGCTTGTCTC
Mouse *Ccl5*	TTTGCCTACCTCTCCCTCG	CGACTGCAAGATTGGAGCACT
Mouse *Ip10*	CCAAGTGCTGCCGTCATTTTC	GGCTCGCAGGGATGATTTCAA
Mouse *β-actin*	AGTGTGACGTTGACATCCGT	GCAGCTCAGTAACAGTCCGC
Mouse let-7a	UGAGGUAGUAGGUUGUAUAGUU	GTGCAGGGTCCGAGGT
Mouse let-7b	UGAGGUAGUAGGUUGUGUGGUU	GTGCAGGGTCCGAGGT
Mouse let-7c	UGAGGUAGUAGGUUGUAUGGUU	GTGCAGGGTCCGAGGT
Mouse let-7d	AGAGGUAGUAGGUUGCAUAGUU	GTGCAGGGTCCGAGGT
Mouse let-7e	UGAGGUAGGAGGUUGUAUAGUU	GTGCAGGGTCCGAGGT
Mouse let-7f	UGAGGUAGUAGAUUGUAUAGUU	GTGCAGGGTCCGAGGT
Mouse let-7g	UGAGGUAGUAGUUUGUACAGUU	GTGCAGGGTCCGAGGT
Mouse let-7i	UGAGGUAGUAGUUUGUGCUGUU	GTGCAGGGTCCGAGGT
Mouse let-7k	UGAGGUAGGAGGUUGUGUG	GTGCAGGGTCCGAGGT
Human *RasGRP1*	CAGGAACTGGTGAAAGCTAAGG	AGTCACGGGCATTGATTTGAG
Human *IL6*	ACTCACCTCTTCAGAACGAATTG	CCATCTTTGGAAGGTTCAGGTTG
Human *TNF*	CCTCTCTCTAATCAGCCCTCTG	GAGGACCTGGGAGTAGATGAG
Human *IL1B*	AGCTACGAATCTCCGACCAC	CGTTATCCCATGTGTCGAAGAA
Human *iNOS*	TTCAGTATCACAACCTCAGCAAG	TGGACCTGCAAGTTAAAATCCC
Human *CCL5*	CCAGCAGTCGTCTTTGTCAC	CTCTGGGTTGGCACACACTT
Human *IP10*	GTGGCATTCAAGGAGTACCTC	TGATGGCCTTCGATTCTGGATT
Human *SOS1*	GAGTGAATCTGCATGTCGGTT	CTCTCATGTTTGGCTCCTACAC
Human *SOS2*	CCGCAGCCTTACGAGTTCTTC	GGATGCACTTGTTCCTGAACC
Human *EGF*	TGGATGTGCTTGATAAGCGG	ACCATGTCCTTTCCAGTGTGT
Human *EGFR*	AGGCACGAGTAACAAGCTCAC	ATGAGGACATAACCAGCCACC
Human *GAPDH*	GGAGCGAGATCCCTCCAAAAT	GGCTGTTGTCATACTTCTCATGG

Supplementary Table 3. Primers used in 3' UTR reporter gene vector construction

Vector	Forward Primer (5'→3')	Reverse Primer (5'→3')
Mouse *Rasgrp1* 3'UTR	TCCAGAATTTCAAGGACCAGAATCT	GTGTATGCCATGGTAATTGCTGTTT
Mouse *Rasgrp1* 3'UTR mut-1#	TCCAGAATTTCAAGGACCAGAATCT	AGAGGCTCTTGATGAGGTCTGAGGT AGCGAACACTGTAGGGATCAGTTTCAC
Mouse *Rasgrp1* 3'UTR mut-2#	GTGAAACTGATCCCTACAGTGTTCGC TACCTCAGACCTCATCAAGAGCCTCT	GTGTATGCCATGGTAATTGCTGTTT
Mouse *Il6* 3'UTR	CGACGCGTGGACCAAGACCATCCAA TTCAT	CCCAAGCTTGGTTTTTAAATATAATATAA TTTAT
Mouse *Il6* 3'UTR mut-1#	CGACGCGTGGACCAAGACCATCCAA TTCAT	ATTTAAATTAGCAATTCATTGACCTAAAC TTATACATTCCAAGAAACCAT
Mouse *Il6* 3'UTR mut-2#	ATGGTTTCTTGGAATGTATAAGTTTAG GTCAATGAATTGCTAATTTAAAT	CCCAAGCTTGGTTTTTAAATATAATATAAT TTAT

Supplementary Table 4. Primers used in RIP analysis for detecting *Il6*

Vector	Forward Primer (5'→3')	Reverse Primer (5'→3')
Primer 1#	ACGATGATGCACTTGCAGAAA	TGACTCCAGCTTATCTCTTGGT
Primer 2#	CCCCAATTTCCAATGCTCTCC	CGCACTAGGTTTGCCGAGTA
