# Supplementary material for: RasGRP1 promotes the acute inflammatory response and restricts inflammation-associated cancer cell growth
Source: Nat Commun. 2022 Nov 16;13:7001. doi: 10.1038/s41467-022-34659-x (PMC9669001; doi:10.1038/s41467-022-34659-x)
Supplement: Supplementary file 3 — Reporting Summary [file 41467_2022_34659_MOESM3_ESM.pdf]

nature portfolio

Corresponding author(s): Songqing Tang

Last updated by author(s): Sep 29, 2022

Reporting Summary

Nature Portfolio wishes to improve the reproducibility of the work that we publish. This form provides structure for consistency and transparency in reporting. For further information on Nature Portfolio policies, see our Editorial Policies and the Editorial Policy Checklist.

Statistics

For all statistical analyses, confirm that the following items are present in the figure legend, table legend, main text, or Methods section.

n/a	Confirmed	
☐	☒	The exact sample size (*n*) for each experimental group/condition, given as a discrete number and unit of measurement
☐	☒	A statement on whether measurements were taken from distinct samples or whether the same sample was measured repeatedly
☐	☒	The statistical test(s) used AND whether they are one- or two-sided<br>*Only common tests should be described solely by name; describe more complex techniques in the Methods section.*
☒	☐	A description of all covariates tested
☒	☐	A description of any assumptions or corrections, such as tests of normality and adjustment for multiple comparisons
☐	☒	A full description of the statistical parameters including central tendency (e.g. means) or other basic estimates (e.g. regression coefficient) AND variation (e.g. standard deviation) or associated estimates of uncertainty (e.g. confidence intervals)
☐	☒	For null hypothesis testing, the test statistic (e.g. *F*, *t*, *r*) with confidence intervals, effect sizes, degrees of freedom and *P* value noted<br>*Give P values as exact values whenever suitable.*
☒	☐	For Bayesian analysis, information on the choice of priors and Markov chain Monte Carlo settings
☒	☐	For hierarchical and complex designs, identification of the appropriate level for tests and full reporting of outcomes
☐	☒	Estimates of effect sizes (e.g. Cohen's *d*, Pearson's *r*), indicating how they were calculated

Our web collection on statistics for biologists contains articles on many of the points above.

Software and code

Policy information about availability of computer code

Data collection	We collected the data of RasGRP1 gene expression on the survival of multiple kinds of cancer patients using the Kaplan-Meier Plotter database (http://kmplot.com/analysis/)
Data analysis	TargetScan Human 7.2 (http://www.targetscan.org/vert_72) was used for predicted microRNA targets.<br>All statistical analyses were performed using SPSS 25.0.<br>Colocalization analyses were performed using Coloc 2.<br>Image analyses were performed using Image-Pro Plus 6.0.

For manuscripts utilizing custom algorithms or software that are central to the research but not yet described in published literature, software must be made available to editors and reviewers. We strongly encourage code deposition in a community repository (e.g. GitHub). See the Nature Portfolio guidelines for submitting code & software for further information.

Data

Policy information about availability of data

All manuscripts must include a data availability statement. This statement should provide the following information, where applicable:

- Accession codes, unique identifiers, or web links for publicly available datasets
- A description of any restrictions on data availability
- For clinical datasets or third party data, please ensure that the statement adheres to our policy

The microarray data generated in thist study has been deposited in the NCBI Gene Expression Omnibus database 57 and is accessible through GEO Series accession number GSE165800 (https://www.ncbi.nlm.nih.gov/geo/query/acc.cgi?acc=GSE165800). All data generated in this study are provided in the Supplementary Information/Source Data file.

Field-specific reporting

Please select the one below that is the best fit for your research. If you are not sure, read the appropriate sections before making your selection.

- [x] Life sciences
- [ ] Behavioural & social sciences
- [ ] Ecological, evolutionary & environmental sciences

For a reference copy of the document with all sections, see nature.com/documents/nr-reporting-summary-flat.pdf

Life sciences study design

All studies must disclose on these points even when the disclosure is negative.

Sample size	We predetermined sample size by SPSS 25.0. (Power: 0.9; Alpha: 0.05; Two-Sided).
Data exclusions	No data were excluded from the analyses.
Replication	All experiments were repeated three times and similar results were obtained in three independent experiments.
Randomization	We used randomization method for the group allocation of all experiments.
Blinding	We were blinded to group allocation during data collection and/or analysis.

Reporting for specific materials, systems and methods

We require information from authors about some types of materials, experimental systems and methods used in many studies. Here, indicate whether each material, system or method listed is relevant to your study. If you are not sure if a list item applies to your research, read the appropriate section before selecting a response.

Materials & experimental systems

n/a	Involved in the study	
	x	Antibodies
	x	Eukaryotic cell lines
x		Palaeontology and archaeology
	x	Animals and other organisms
	x	Human research participants
x		Clinical data
x		Dual use research of concern

Methods

n/a	Involved in the study	
x		ChIP-seq
x		Flow cytometry
x		MRI-based neuroimaging

Antibodies

Antibodies used

Abs against total and phosphorylated forms of ERK1/2 (Thr202/Tyr204) (4695,9101), JNK1/2 (Thr183/Tyr185) (4668,9252), P38 (Thr180/Tyr182) (9212,9215), IKKa/b (Ser176/180) (2697), Akt (Ser473) (4060,9272), IkBa (Ser32/36) (4814,9246), IL-6 (12912), F4/80 (30325), Ki-67 (9129) were from Cell Signaling Technology (Beverly,MA). Antibodies specific to RasGRP1(ab37927), Cy3 (ab52060) and GAPDH (ab128915) were from Abcam Inc. (Cambridge,MA). Ab for b-actin (A1978) was from Sigma. HRP-labeled goat anti-rabbit IgG (AP132P) was from Meck Millipore.

Validation

ERK1/2 (Thr202/Tyr204) (4695): Application: WB, IP, IHC, IF, F Reactivity: H M R Hm Mk Mi Dm Z B Dg Pg Ce
p-ERK1/2 (Thr202/Tyr204) (9101): Application: WB, IP, IF, F Reactivity: H M R Hm Mk Mi Dm Z B Pg Ce
p-JNK1/2 (Thr183/Tyr185) (4668): Application: WB, IP, IHC, Reactivity: H M R Dm Sc
JNK1/2 (Thr183/Tyr185) (9252): Application: WB Reactivity: H M R Hm Mk Z B Sc
P38 (Thr180/Tyr182) (9212): Application: WB, IHC, IF, F Reactivity: H M R Mk GP
p-P38 (Thr180/Tyr182) (9215): Application: WB, IF, F Reactivity: H M R Mk Dm Pg Sc
p-IKKa/b (Ser176/180) (2697): Application: WB, IHC, F Reactivity: H M R Mk
p-Akt (Ser473) (4060): Application: WB, IP, IHC, IF, F Reactivity: H M R Hm Mk Dm Z B
Akt (Ser473) (9272): Application: WB, IP, IF, F Reactivity: H M R Hm Mk C Dm B Dg Pg GP
IkBa (Ser32/36) (4814): Application: WB, IP, IHC, IF, F Reactivity: H M R Mk B Pg
p-IkBa (Ser32/36) (9246): Application: WB Reactivity: H M R Mk
IL-6 (12912): Application: WB, IP, IF, F Reactivity: M
F4/80 (30325): Application: WB, IP, IF Reactivity: M
Ki-67 (9129): Application: IF, F Reactivity: H M R
RasGRP1: Application: WB and IHC-P, Reactivity: Mouse, Rat, Human, Xenopus laevis.
Cy3: Application: Dot, ELISA, ICC, ICC/IF, IP; Reactivity: Species independent
GAPDH (ab128915): Application: Flow Cyt (Intra), ICC/IF, IHC-P, IP, WB Reactivity: Human, African green monkey
b-actin (A1978): Application: ARR, ICC, IF, IHC (f), WB Reactivity: pig, Hirudo medicinalis, bovine, rat, canine, feline, human, rabbit,

	carp, mouse, guinea pig, chicken, sheep

Eukaryotic cell lines

Policy information about cell lines

Cell line source(s)	HEK293 cells, HEK293t cells, Huh7 cells and RAW264.7 cells were obtained from the American Type Culture Collection (Manassas, VA) .
Authentication	None of the cell lines used were authenticated.
Mycoplasma contamination	All cell lines used in this study tested negative for mycoplasma contamination.
Commonly misidentified lines (See ICLAC register)	There was no commonly misidentified cell lines used in this study.

Animals and other organisms

Policy information about studies involving animals; ARRIVE guidelines recommended for reporting animal research

Laboratory animals	Wild-type or IL6-knockout C57BL/6 mice (6–8 weeks of age, male) were used in this study.
Wild animals	This study did not involve wild animals.
Field-collected samples	This study did not involve samples collected from the field.
Ethics oversight	This study was approved by the Animal Care and Use Committee of the School of Medicine, Zhejiang University.

Note that full information on the approval of the study protocol must also be provided in the manuscript.

Human research participants

Policy information about studies involving human research participants

Population characteristics	Colon cancer patients: 3 participants (66 years, female; 73 years, male; 78 years, male)<br>Gastric cancer patients: 3 participants (62 years, male; 63 years, female; 81 years, male)
Recruitment	All participants were randomly recruited.
Ethics oversight	This study was approved by the institutional review boards of Hunan Cancer Hospital.

Note that full information on the approval of the study protocol must also be provided in the manuscript.
